# A High-Throughput Phenotyping Pipeline for Image Processing and Functional Growth Curve Analysis

**DOI:** 10.34133/2020/7481687

**Published:** 2020-07-14

**Authors:** Ronghao Wang, Yumou Qiu, Yuzhen Zhou, Zhikai Liang, James C. Schnable

**Affiliations:** ^1^Department of Statistics, University of Nebraska-Lincoln, Lincoln 68503, USA; ^2^Department of Statistics, Iowa State University, Ames 50011, USA; ^3^Department of Plant and Microbial Biology, University of Minnesota, Saint Paul, MN 55108, USA; ^4^Center for Plant Science Innovation, Department of Agronomy and Horticulture, University of Nebraska-Lincoln, Lincoln 68503, USA

## Abstract

High-throughput phenotyping system has become more and more popular in plant science research. The data analysis for such a system typically involves two steps: plant feature extraction through image processing and statistical analysis for the extracted features. The current approach is to perform those two steps on different platforms. We develop the package “implant” in R for both robust feature extraction and functional data analysis. For image processing, the “implant” package provides methods including thresholding, hidden Markov random field model, and morphological operations. For statistical analysis, this package can produce nonparametric curve fitting with its confidence region for plant growth. A functional ANOVA model to test for the treatment and genotype effects on the plant growth dynamics is also provided.

## 1. Introduction

High-throughput phenotyping is a newly emerging technique in the plant science research. Many automated systems have been constructed both in the greenhouse and field to study plant features [1–3]). One of the main innovations is to use automated cameras to take raw images for plants. Several types of high-resolution images, including RGB, infrared, flourescence, and hyperspectral, are recorded for a large number of plants at designed time points. From the images, we are able to process and extract useful phenotypical features, such as plant height, width, and size. Compared to the traditional methods, the high-throughput system is able to provide the plant features of interest in a more efficient, accurate and nondestructive way.

In order to extract the plant traits, segmentation for parts of a plant or the whole plant is necessary. Thresholding is the simplest and the most commonly used method for image segmentation [4], which separates the image into foreground and background classes by a cut-off value on the pixel intensities. Based on the thresholding methods, several platforms have been developed for the analysis of high-throughput plant phenotyping, including HTPheno [5], Image Harvest [6], and PlantCV [7]. Those software have admitted procedures in processing plant images to extract phenotypical features. However, these platforms solely focus on image processing. There is a lack of functionality on statistical modeling and inference for plant growth.

*K*-means clustering algorithm [8] is also well known for image segmentation, which assigns pixels into subgroups so that the within-group variation of pixel intensity is minimized. When the number of clusters is given, the *K*-means method is free of tuning parameter selection. The hidden Markov random field model (HMRF) [9] can be applied to refine the segmentation result from *K*-means clustering and thresholding. HMRF is a hierarchical model with a hidden layer of Markov random field to model the class label of each pixel, which captures the spatial dependence of pixels to their neighborhood. As the thresholding and *K*-means methods ignore the spatial structure of an image, the HMRF model is able to provide a more accurate classification of pixels by incorporating their neighborhood class information.

Given an accurate segmentation, the measurements of phenotypical traits can be extracted from the images. These numerical measurements can be used for analyzing genotype and treatment effects on the plant growth over time. In a traditional growth curve analysis, the approach of a pointwise analysis of variance (ANOVA) is applied at each measurement time point. However, this approach analyzes each time point separately and thus cannot reflect the dynamics of plant growth. Parametric modeling for the growth curve is another popular tool. However, fitting the parametric models requires measurements of the plant traits over the whole growth stage which may not be available for some experiments, and the temporal dependence of the data is usually ignored in this approach. Functional ANOVA [10] is a recent nonparametric method for analyzing plant traits collected over time [11]. Instead of parametric regression, smoothing splines [10, 12] or local polynomial regression [13] is used to estimate the plant growth. This approach is nonparametric, fully data driven, and adaptive to temporal dependence of the data. Despite those advantages, the implementation of functional ANOVA for plant phenotyping data [11] is nontrivial. The current R package “fda” [14] for functional data analysis is complicated, and it is difficult to use for nonstatisticians. There is no computation guidance of functional ANOVA on studying plant growth.

**Figure 1:**
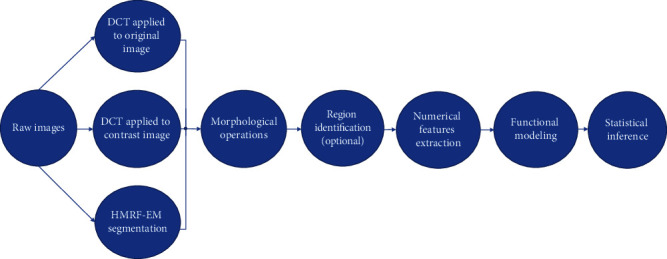
Flow chart of the proposed “implant” pipeline. In the first step of segmentation, multiple methods could be jointly applied and the common plant area is considered to be the final segmentation.

To respond to the needs of data analysis for the high-throughput phenotyping systems, we develop an R package “implant” that involves both image processing and functional data analysis for the extracted traits. The scope of this paper is to provide an easy-to-use pipeline to analyze high-throughput phenotyping data, from the raw images to statistical analysis. Compared to [11] that mainly focuses on introducing the methodology of nonparametric curve fitting, the proposed package provides a user friendly computation tool, which allows plant scientists to easily conduct functional data analysis for plant growth dynamics. Our package also provides the confidence regions for the time-varying regression coefficients, which is not thoroughly discussed in Xu et al. [11]. The flow chart in Figure 1 illustrates the main steps of this pipeline. In the first step, plant segmentation is done by double-criteria thresholding (DCT) or HMRF methods. Notice that if the image of an empty pot is available, DCT can be applied on the contrast image between the plant and the empty pot as demonstrated in Figures 1(a)–1(c).

**Figure 2:**
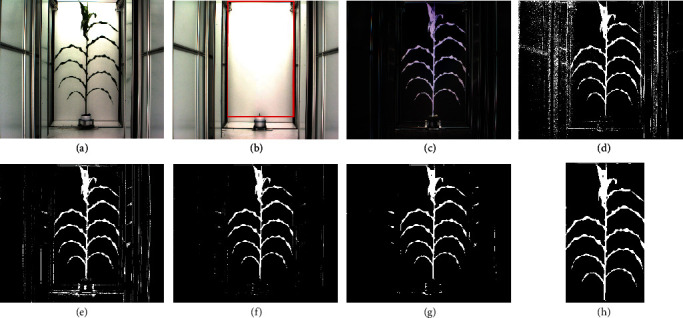
(a) Original plant image. (b) Original empty pot image; the red square is the identified region of interest by the functions “ColorB” and “ColorG.” (c) Contrast of (a) and (b). (d) Segmented image of (a) using DCT. (e) Segmented image of (c) using DCT. (f) Intersection of (d) and (e). (g) Dilated-eroded-eroded-dilated image of (f). (h) Final segmented image by identifying the region of interest.

Figure 2 In the second step, morphological erosion and dilation operations [15] and plant region identification can be applied to refine the segmentation. Then, plant traits are calculated based on the segmented image. In the last step, functional data analysis and statistical inference are conducted on the extracted traits. The pipeline is able to estimate both the main and interaction effects on the plant growth, provide confidence regions for the effect curves, and deal with irregular observation time points. Those confidence regions can demonstrate the statistical significance for the treatment and genotype effects over time. A real data example is provided in Implementation and Results to illustrate the utility of our package.

## 2. Methods

In this section, we introduce the hidden Markov random field model for image segmentation and the functional ANOVA for growth curve analysis.

### 2.1. Hidden Markov Random Field Model

The hidden Markov random field (HMRF) model is a hierarchical model with an unobserved layer for the pixel class and an observed layer for the pixel intensity given its class. The hidden layer of the pixel class is modeled by a Markov random field, where the probability of a pixel from the plant category depends on the classes of its neighborhood pixels. As the plant pixel is more likely to be surrounded by plants, this transition probability matrix models the spatial dependence of the pixel classes. We assume that the pixel intensity follows a normal distribution where its mean and variance are determined by the class of this pixel. And, the joint distribution of the unobservable classes for all the pixels follows the Gibbs distribution, according to the Hammersley-Clifford Theorem [16]. The aim of this method is that for each pixel, given the observed pixel intensity, we predict its class label by maximizing the probability that the pixel is classified into this class.

We use the relative green intensity as our response variable. In order to fit the HMRF model, we apply the expectation maximization (EM) algorithm [17, 18]. By using the segmentation results obtained by *K*-means clustering as the initial class label for the EM algorithm, we iteratively find the maximal likelihood estimators for the mean and variance of the relative green intensities for the plant class and the background class. Then, the class label of each pixel is predicted as the class with higher posterior probability of the pixel belonging to given the observed intensities.

### 2.2. Functional ANOVA

Phenotypical traits extracted from the segmented images can be used to build functional data models and draw statistical inference for the plant growth dynamics [10, 11]. We consider functional data analysis for one type of the extracted traits, denoted by *y*_*i*_(*t*) as the measurement of the *i*th plant at time *t*. Since the high-throughput measurements for each plant are relatively dense over time and the plant growth curve is smooth, we directly use the extracted trait as the response instead of its smoothed values. Suppose the trait is potentially affected by *q* factors. With the number of levels of the *j*th factor denoted by *ℓ*_*j*_, we define **X**_*ij*_ = (*x*_*ij*2_, ⋯,*x*_*ijℓ*_*j*__)^*T*^ as the categorical indicators of the *j*th factor of the *i*th plant. Specifically, *x*_*ijk*_ is set to one if the *j*th factor of the *i*th plant has level “*k*”; otherwise, *x*_*ijk*_ = 0. With the Kronecker product of matrices denoted by ⊗, a functional multiway ANOVA model with interactions can be written in the following form:

(1) $$\begin{array}{c} y_{i}\left(t\right)=\mu \left(t\right)+X_{i1}^{T}a_{1}\left(t\right)+X_{i2}^{T}a_{2}\left(t\right)+\cdots +X_{iq}^{T}a_{q}\left(t\right)+\left(X_{i1}^{T}⊗X_{i2}^{T}\right)a_{1,2}\left(t\right)+\cdots +\left(X_{iq-1}^{T}⊗X_{iq}^{T}\right)a_{q-1,q}\left(t\right)+\varepsilon_{i}\left(t\right), \end{array}$$

where **a**_*j*_(*t*) = (*a*_*j*2_(*t*), ⋯,*a*_*jℓ*_*j*__(*t*))^*T*^ values are the treatment effect functions of the *j*th factor with dimension *ℓ*_*j*_ − 1, **a**_*j*_1_,*j*_2__(*t*) values are the pairwise interaction effect functions between factors *j*_1_ and *j*_2_ with dimension (*ℓ*_*j*_1__ − 1)(*ℓ*_*j*_2__ − 1), and *ε*_*i*_(*t*) values are temporal dependent random processes with zero means. We have implemented this multifactor model (*q* ≥ 2) in our package such that researchers can specify the main and interaction effects as needed. A real data example without interaction is illustrated in the next section, and another example with interaction is provided in the user guide.

**Figure 3:**
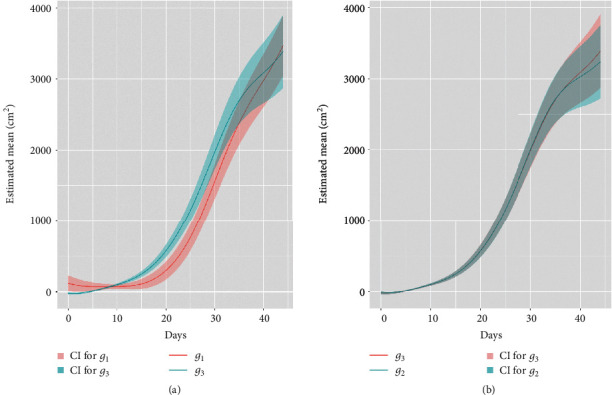
(a) 95% confidence regions for the average plant size of genotypes 1 and 3 over the three blocks. (b) 95% confidence regions for the average plant size of genotypes 2 and 3 over the three blocks.

We approximate all of the coefficient functions with rank *K* B-spline expansion. For example, *a*_*jk*_(*t*) = ∑_*v*=1_^*K*^*β*_*jk*,*v*_*B*_*d*,*v*_(*t*), where {*B*_*d*,*v*_(*t*)}_*v*=1_^*K*^ values are order *d* B-spline basis functions, and {*β*_*jk*,*v*_}_*v*=1_^*K*^ values are coefficients of the basis functions. The rank *K* of the B-spline basis functions is equal to the order (degree + 1) of the spline plus the number of interior knots. To avoid overfitting, we choose the rank that is less than half of the number of observation time points *m*. Since the growth curves of plants are relatively smooth as shown in Figure 3, we use splines with degree 3 and *m*/2 − 4 equally spaced interior knots. Then, the estimator $\overset{\wedge }{\beta }$ for the spline coefficients can be found explicitly via the penalized smoothing splines. We penalize the *L*_2_ norm of the second derivatives of the spline expansion functions and choose a common smoothing parameter *λ* by the generalized crossvalidation (GCV). We develop the function “fanova” in our package to implement the estimation procedure. It turns out that $\overset{\wedge }{\beta }$ is a linear estimator of the response *y*_*i*_(*t*). Given the sample covariance of the response *y*_*i*_(*t*), it is straightforward to estimate the covariance of $\overset{\wedge }{\beta }$ and hence the covariance of ${\overset{\wedge }{a}}_{jk}$ based on the spline expansion. Confidence regions of the treatment effects can be constructed accordingly, which can be obtained by the functions “CI_contrast” and “CI” in the package.

## 3. Implementation and Results

In this section, we illustrate the implementation of our “implant” package by a maize experiment conducted at the University of Nebraska-Lincoln (UNL) Greenhouse Innovation Center. The package, documentation, and user guide are available online at https://github.com/rwang14/implant. Detailed descriptions for the methods are presented in Methods.

### 3.1. Experiment

The experiment involved 420 maize plants with 140 different genotypes. There were three replicates for each genotype. The pots in the greenhouse were divided into three blocks based on the layout of the belt conveyor system. We conducted the randomized complete block design (RCBD) such that each of the 140 genotypes was randomly located within a block and the three replicates of the same genotype were assigned to different blocks. Each plant was imaged about every two or three days from May to July, and the imaging time points were irregular due to the large number of plants in the experiment.

### 3.2. Image Processing

#### 3.2.1. Image Segmentation Using DCT

Figure 2 shows the general process of the double-criteria thresholding (DCT) for one of the plant images from the experiment. Here, Figures 2(a) and 2(b) are the RGB maize image and the empty pot image, respectively. Figure 2(d) is obtained by the function:

(2) $$\begin{array}{c} imageB=imageBinary\left(original\_image,weight=c\left(-1,2,-1\right),threshold1=30/255,threshold2=0.02\right), \end{array}$$

where “threshold1” is applied to the sum of the RGB intensities, and “threshold2” is applied on the green contrast intensity by the specified weight in the function [19]. The two thresholds are to delete the black pixels and to segment the plant green pixels, respectively. We choose a small level 0.02 for the second threshold to retain most part of the plant. The background noises in Figure 2(d) can be much reduced by applying the DCT procedure on the contrast image in Figure 2(c) resulting from the difference between Figures 2(a) and 2(b). The image in Figure 2(e) is obtained by setting “threshold1 = 0.7,” “threshold2 = 0,” and “weight = c(1, −2, 1)” in the “imageBinary” function for Figure 2(c). Then, we take the intersection between Figures 2(d) and 2(e) to obtain Figure 2(f). As we observed from Figure 2(f), most of the background noises are eliminated and the plant body is segmented well. Those thresholding parameters work consistently well over the whole experiments for the UNL greenhouse. The double-criteria procedure makes the results less sensitive to the threshold levels than the procedure using only one criterion. Though for a different system, those parameters need to be properly tuned for good segmentation results. In the case of no empty pot images, we should set a more restrictive threshold for the green contrast intensities.

Morphological operations can be applied on the thresholding results to further reduce the segmentation errors (see Figure 2(g)), which can be performed by the “dilation” and “erosion” functions in our packages as follows:

(3) $$\begin{array}{c} imageBD=dilation\left(imageB,mask=matrix\left(1,5,5\right)\right),\\ imageBDE=erosion\left(imageBD,mask=matrix\left(1,5,5\right)\right).\\ imageBDEE=erosion\left(imageBDE,mask=matrix\left(1,3,3\right)\right),\\ imageBDEED=dilation\left(imageBDEE,mask=matrix\left(1,3,3\right)\right), \end{array}$$

where “mask” is a structuring matrix specifying the neighborhood structure of a pixel [15]. The dilation operator is applied to a binary image to enlarge the boundaries of the segmented object and fill in the holes within the object. Erosion is the opposite operator to dilation, which erodes away the boundaries of the segmented object. We call dilation followed by erosion as a morphological closing operation, and erosion followed by dilation as a morphological opening operation.

Region of interest can be automatically identified by some specific characteristics on the background of an imaging system. For the UNL greenhouse, we can identify the inner black bars and the border top of the pot to obtain the region of interest for the plants; see the red rectangle in Figure 2(b). Notice that this identification strategy is for the images from the UNL greenhouse system only. Different systems need different strategies for locating the region of interest. It is worth mentioning that although identifying the region of interest can help us easily remove most of the background noises, parts of the plants might be lost as well (see Figure 2(h) as the chopped image by the identified region in Figure 2(b)).

#### 3.2.2. Image Segmentation Using HMRF

**Figure 4:**
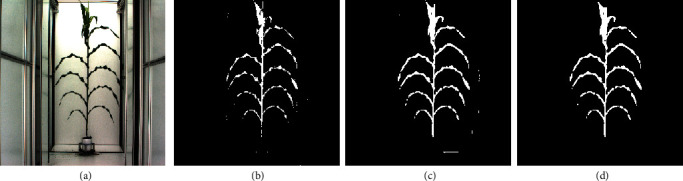
(a) Original image. (b) Initial classification using *K*-means (*K* = 2) on relative green intensity *G*/(*R* + *G* + *B*). (c) Segmentation result using HMRF. (d) Applying morphological closing and opening to (c).

The segmentation method by the HMRF model is also provided in the package. Compared to the former thresholding procedure, the HMRF model is data driven and free of tuning parameter selection.  Figure 4(c) shows the segmentation result by the HMRF model with initial class assignment by the *K*-means clustering algorithm on relative green intensity *G*/(*R* + *G* + *B*) with *K* = 2. From Figure 4(c), we see that the HMRF model provides a quite good segmentation for the plant with few classification errors. Comparing to the *K*-means result in Figure 4(b), the HMRF is able to fill in the missing plant pixels by using their neighborhood class information. Comparing the thresholding result in Figure 2(f), the result from the HMRF approach eliminates most of the background noises. This method is implemented by the function “HMRF” in the package as

(4) $$\begin{array}{c} HMRF\left(X,Y,\cdots \right)\text{\$}image_{matrix}, \end{array}$$

where *X* is a matrix of initial class labels (for example, results from *K*-means clustering), and *Y* is a matrix of relative green intensities. Description on other arguments of this function can be found in the help documentation of the “implant” package. Morphological operations can be applied on the segmented result from HMRF model, see Figure 4(d) which shows a better segmentation than the morphological operations on the thresholding result in Figure 2(g). The HMRF method can generally get a good segmentation result without identifying the region of interest, which broadens the scope of its application. Moreover, it can be used to refine the segmentation results obtained by other methods.

## 4. Plant Feature Extraction

Based on the segmented images, we can extract the phenotypical features of plants. Given the information of the pixel size in millimeters, we can obtain plant height, width, and size using the functions.

(5) $$\begin{array}{c} extract\_pheno\left(processed\_image,Xsize=1,Ysize=1,\cdots \right), \end{array}$$

where “processed_image” is the segmented image of a plant as in Figures 2(h) and 4(d), and “Xsize” and “Ysize” are the actual horizontal and vertical lengths of one pixel.

## 5. Plant Growth Dynamics Analysis

Based on the extracted traits, we can study the treatment and genotype effects on plant growth. Let *t*_*ij*_ be the *j*th observation time of the *i*th plant, and let *y*_*i*_(*t*_*ij*_) denote a specific trait of the *i*th plant measured at time *t*_*ij*_. Note that there are 140 different genotypes with one replicate in each of the 3 blocks in this study. Let {*G*_*ik*_}_*k*=2_^140^ be the genotype indicators for the *i*th plant, where *G*_*ik*_ = 1 if the *i*th plant has the *k*th genotype, and *G*_*ik*_ = 0 otherwise. Similarly, let {*P*_*ik*_}_2_^3^ be the block indicators. The first genotype and the first block are treated as the baseline. The functional ANOVA model is

(6) $$\begin{array}{c} y_{i}\left(t_{ij}\right)=\mu \left(t_{ij}\right)+\overset{140}{\underset{k=2}{\sum }}G_{ik}g_{k}\left(t_{ij}\right)+\overset{3}{\underset{k=2}{\sum }}P_{ik}p_{k}\left(t_{ij}\right)+\varepsilon_{i}\left(t_{ij}\right), \end{array}$$

where *μ*(*t*), *g*_*k*_(*t*), and *p*_*k*_(*t*) are the intercept, genotype effect, and block effect functions, respectively. The regression error *ε*_*i*_(*t*) is modeled by a temporal dependent random process with mean zero (see Methods for more details).

The regression coefficient curves *μ*(*t*), *g*_*k*_(*t*), and *p*_*k*_(*t*) can be estimated by the function “fanova” in our package,

(7) $$\begin{array}{c} fit=fanova\left(Y.na.mat,X,formula,\cdots \right), \end{array}$$

where *X* specifies levels of genotype and block factors, Y.na.mat is the matrix of the extracted traits, and formula specifies the model, namely, Equation (6) in this example. Then, we can construct the confidence regions for the significance of the treatment and genotype effects by the functions:

(8) $$\begin{array}{c} CI\_contrast\left(fit,j1,j2,\cdots \right),\\ CI\left(fit,L,\cdots \right), \end{array}$$

where fit is the fitted model from the output of *fanova*. The first function provides the confidence regions for the comparison of two treatments/genotypes, where *j*_1_ and *j*_2_ specify the columns of the design matrix corresponding to the treatments/genotypes of interest. The second one offers the confidence regions for general linear combination of coefficients, where *L* is a contrast vector under model (8). This includes estimating the average growth curve of a particular genotype over all the blocks.

**Figure 5:**
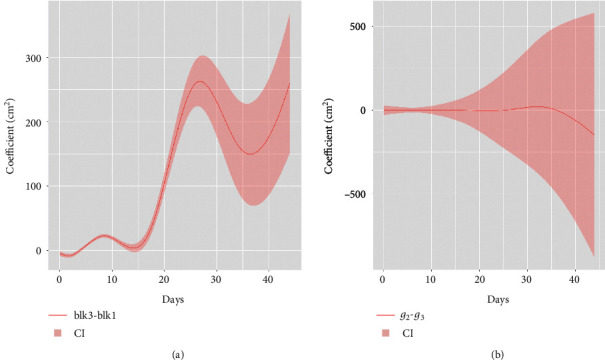
(a) 95% confidence region for the block effect between block 3 and block 1. (b) 95% confidence region for the genotype effect between genotype 2 and genotype 3.

As an example, we consider plant size in this study. The confidence region of the block effect function *p*_3_(*t*) infers whether there is a significant difference in plant size between block 1 and block 3, given the same plant genotype. By choosing *j*_1_ = 142 (the last two columns in the design matrix correspond to blocks 2 and 3, respectively) and *j*_2_ = 1 (intercept) in the function “CI_contrast,” Figure 5(a) shows the 95% confidence region of *p*_3_(*t*) from day 1 to day 44, which shows a significant positive effect of block 3 compared to block 1 especially in the later dates. Similarly, we can construct the confidence regions for *g*_2_(*t*) − *g*_3_(*t*) by setting *j*_1_ = 2 and *j*_2_ = 3, which test for the significance of the difference between the 2^nd^ and the 3^rd^ genotypes. Figure 5(b) shows the 95% confidence region for the genotype effect, which is not significant over the whole time course.

Under model (6), when the contrast vector *L* in the function CI takes (1, 0, ⋯, 0, 1/3, 1/3) and (1, 0, 1, 0, ⋯, 0, 1/3, 1/3), respectively, we obtain the estimated growth curves for genotypes 1 and 3 averaging over the three blocks with their 95% confidence regions, see Figure 3(a). We see that genotype 3 significantly grew faster than genotype 1 from day 10 to day 30. Similarly, Figure 3(b) shows the growth curves for genotypes 2 and 3 with their 95% confidence regions, which overlap with each other and demonstrate no significant difference between those two genotypes. This coincides with the result from Figure 5(b).

## 6. Discussion

In this paper, we developed a comprehensive pipeline for analyzing high-throughput plant phenotyping data that includes RGB image preprocessing, plant feature extraction, and functional data modeling and inference for growth curve dynamics. In recent literature, there has been an increasing trend of using deep convolutional neural networks (CNN) to extract plant traits from images, see Lu et al. [20] and Miao et al. [21] for counting tassels and leaves of maize plants, respectively; Pound et al.[22] for identifying wheat images containing spikes and spikelets as well as counting their numbers; and Aich et al. [23] for estimating emergence and biomass of wheat plants. Compared to the traditional approaches used in the proposed pipeline (image segmentation by thresholding + feature calculation + statistical modeling), the deep learning methods are able to work under an unconstrained field environment, where the thresholding method may not give a reliable and robust separation of the plants from backgrounds. However, such deep neural nets typically have a vast number of parameters. A sufficiently large training sample and intensive computation are required to train those models. Preparing the training data is both time and labor consuming. As a comparison, thresholding methods are computationally efficient and easy to implement without a training set, and they can give accurate segmentation for plants under homogeneous backgrounds, as in a greenhouse environment. More importantly, the statistical analysis is able to model and study the biological mechanism on the plant growth, which is an advantage over the ‘black box' methods.

In a recent work, Adams et al. [24] proposed a neural network model trained on over half million pixels from maize images for plant segmentation. Although, it is more time consuming than the thresholding method in computation, this method is able to achieve robust plant segmentation under noisy backgrounds. We will include such neural network methods in the next version of the “implant” package to improve the current functionality for field images.

Beside the RGB images, the UNL greenhouse also takes the hyperspectral images for every plant. Compared to RGB images which only have three channels, the hyperspectral images record the pixel intensities at every 5 nm over the whole spectrum, which contain more information than RGB images. The hyperspectral images can be used to separate plant organs and predict chemical concentration within a plant [19]. In future works, we will extend the HMRF model and functional ANOVA to hyperspectral images for studying traits from different plant organs.

## Data Availability

Data will be made available on request.
